# Association of psychosocial and perceived environmental factors with park-based physical activity among elderly in two cities in China and Germany

**DOI:** 10.1186/s12889-019-8140-z

**Published:** 2020-01-14

**Authors:** Petra Wagner, Yan Ping Duan, Ru Zhang, Hagen Wulff, Walter Brehm

**Affiliations:** 1 Institute for Execise and Public Health, Leipzig University, Jahnallee 59, 04155 Leipzig, Germany; 2 Department of Sport and Physical Education, Hong Kong Baptist University, 8 On Muk Street, Shek Mun, Shatin, Hong Kong China; 3 Department of Sports Science & Physical Education, The Chinese University of Hong Kong, G/F, Kwok Sports Building, Shatin, Hong Kong China; 4 Institute for Execise and Public Health, Leipzig University, Jahnallee 59, 04155 Leipzig, Germany; 5 Institute of Sport Science, University of Bayreuth, 95447 Bayreuth, Germany

**Keywords:** Urban Park, Park-based physical activity, Psychosocial and perceived environmental factors, Cities, Elderly, Mixed-culture sample

## Abstract

**Background:**

Urban parks play an important role in promoting physical activity (PA) among adults and especially among older city residents. According to the socioecological approach the association of physical environments and psychosocial factors in the context of park-based PA of elderly have not been systematically examined until now, let alone the relevance of the city (urban area) on a cross-cultural level. This study investigated selected aspects of (1) the association of psychosocial and park environmental factors with park-based physical activity (PBPA) of older people; and (2) the moderating effect of city on the association of these factors with PBPA.

**Methods:**

A face-to-face survey was conducted of a mixed-culture sample from different urban surroundings in Hong Kong (HK) and Leipzig (L). In six parks of each city physically active elderly (> = 60 years; HK: *n* = 306; L: *n* = 311) were recruited. Multiple linear regressions were used to analyse the association between psychosocial factors and perceived environmental factors with PBPA and the moderating effect of city.

**Results:**

Controlled for demographic variables, all other psychosocial factors were significantly related to PBPA, except social support. In terms of environmental factors, PBPA was positively associated with safety, attractiveness, features and negatively associated with park time distance. Controlled for demographic variables, psychosocial and environmental factors, the moderating effect of city on the associations of park features and park time distance with PBPA was not significant in HK. In contrast, there was a significant positive relationship for park features and a negative relationship for park time distance with PBPA in L.

**Conclusions:**

Psychosocial and perceived environmental factors significantly influence PBPA of older people. City moderates the associations of these factors and independently contributes to park-based PA of the elderly. The different interactions of environmental factors and urban area for PBPA of elderly can support policy makers on the municipal level in choosing adequate strategies for promoting PA of older people in parks.

## Background

Parks have the potential to contribute significantly to the PA of the urban population. A positive relationship between the number of urban parks and PA was found by Sallis, Cervero, Ascher et al. [1] in a study of 14 cities worldwide. Due to free and accessible PA areas, parks have widely been recognized as key environmental sites where individuals can engage in a variety of recreational activities with health benefits in daily life [2].

On the other side, urban parks are still not well utilized for PA by park visitors and residents of surrounding neighborhoods. Findings from park use research have revealed that more than half of city inhabitants never visit parks for active or passive activities during a typical week [3]. Less than one third of surveyed or observed park visitors engage in PBPA [4]. But with respect to “healthy ageing” and “active ageing” [5], urban parks have been recognized as important PA places for senior city residents [6] to increase health-related benefits [7, 8]. Although especially elderly make up at least 20% of the population in many countries, in recent studies of park use they are underrepresented with no more than 5% [4, 9, 10].

In order to promote PA of elderly in parks, the associations of possible factors with PBPA have to be known. According to the socio-ecological approach, older adults’ perceptions of the park environment and psychosocial characteristics could influence their PBPA [1]. PA of elderly is, according to Kerr [11], characterized by low-intensity leisure and day-to-day activities, sport activities and transportation activities, depending on a particular degree of demographic factors such as age, gender or family status [12–14]. Furthermore, psychosocial factors such as self-efficacy, perceived barriers as well as the benefits of PA, enjoyment of PA or social support significantly influence PA behavior [5, 12, 15]. Subjective perceptions of the environment also play a significant role for PA [16]. For older adults, the sense of security with regard to their own body and the environment seem to play another fundamental role. Further relevant factors, according to Sallis et al. [1], are the perceived comfort and attractiveness of environmental conditions. These aspects concern for example the design and use of the transport infrastructure [17] such as walkways, railings and rest stops. Other studies of PBPA, but not with respect to the elderly, revealed that perceived cleanliness in parks was negatively related to PA [18]. Ries et al. [19] and Lackey et al. [20] also posited that perceived access to parks was associated with PBPA. Although associations between physical environments and psychosocial factors are supported for recreational walking [21, 22], active transport [15], and overall PA [15, 22] of older adults, the relationships have not been systematically investigated in the context of PBPA, let alone for PA of the elderly and intercultural comparison [18]. Based on the results of various systematic reviews of PA correlates in older adults [22, 23], studies are needed to assess the association between specific environmental and personal characteristics with older adults’ active park use.

Regarding a socio-ecological approach, physical environmental conditions of urban areas also influence activity behavior [24, 25]. According to Yen et al. [25], the residential area with its various movement areas such as parks, pedestrian and cycle paths, their furnishing or design as well as the presence of further infrastructure (traffic, medical care, shopping, sports facilities) influence the activity behavior of elderly. But most empirical studies on PBPA and psychosocial as well as environmental correlates were conducted in a single region with similar urban conditions [26]. However, sample-specific variations in the findings on PBPA of elderly and their psychosocial and environmental characteristics can be identified using data from different geographical regions with different urban conditions (e.g. built environment, population density) [27, 28].

Thus, based on the reported results and a socio-ecological approach, the objective of the study was to investigate (1) the association of selected psychosocial and perceived park environmental factors with PBPA of older people from Hong Kong in China and Leipzig in Germany; (2) the moderating effect of city (urban area) on the associations of these factors with PBPA.

## Methods

### Selection of cities, parks and survey participants

In this survey, two cities were selected to represent different urban conditions: Hong Kong in China and Leipzig in Germany. Hong Kong has 7.35 million inhabitants, 23% of whom are elderly aged 60 and above. The city has a high density of buildings and population (6958 inhabitants/km^2^). 7.07 million people live in residential high-rises. The size of the 31 urban parks in Hong Kong ranges from 1.76–22.00 ha (mean size: 8.43 ha). Leipzig has 0.56 million inhabitants, 26% of whom are older people aged 60 and above. The city has a low density of buildings and population (1882 inhabitants/km^2^) and very few high-rise residential buildings. The size of the 32 urban parks in Leipzig ranges from 0.40–42.40 ha (mean size: 11.52 ha).

The parks were selected in both cities on the basis of the same criteria. As study settings, the parks had to be accessible, located at different geographical regions, of different sizes and with activity areas [17, 29, 30]. In addition, parks were excluded if they were under construction or renovation during the study period [17]. To balance funding, output precision, and time cost, six parks were included in the study from each city. A small and a large park were selected from each of the three Hong Kong regions. Finally, the survey was conducted in Victoria Park (19.00 ha), Chai Wan Park (7.13 ha), Lai Chi Kok Park (17.65 ha), Shek Kip Mei Park (8.00 ha), Shing Mun Valley Park (10.73 ha) and Tsuen Wan Riviera Park (4.50 ha). In Leipzig, two parks in the city center were selected (Friedenspark: 17.00 ha; Clara-Zetkin-Park: 42.40 ha) and the remaining four parks from city districts in the east (Stadtteilpark Rabet: 5.80 ha), west (Volkspark Kleinzschocher: 40.00 ha), south (Lene-Voigt-Park: 5.60 ha) and north (Arthur-Brettschneider-Park: 7.30 ha).

Active adults aged 60 or above who engaged in PA at low, moderate or vigorous intensity [31] were targeted as survey participants with 60 participants in each park of each city. To have a balanced sample, the older adults were randomly asked to participate in our study from the busiest and least-busy active spaces in the six Hong Kong and Leipzig parks. A total of 720 active older adults (360 in Hong Kong and 360 in Leipzig) were invited to participate in the survey and informed about the purpose of the study with a written informed consent form. Among them, 617 older adults in Hong Kong (*n* = 306, age range: 60–88 years, Mean _age_ = 69.96, *SD* = 6.81) and Leipzig (*n* = 311, age range: 60–92, Mean _age_ = 72.06, *SD* = 6.78) accepted the invitation and completed the survey taking approximately 20–25 min. The data were collected on two weekdays and both weekend days in 1 week in Autumn 2014 and in Spring 2015. The same four trained interviewers carried out the data collection in HK and L respectively.

The sample in Hong Kong (HK) was different from the sample in Leipzig (L) concerning age (*t* (609) = 3.82, *p* < .001), gender (females _HK_ = 46.7%, females _L_ = 58.5%; *χ*^*2*^ (1) = 8.60, *p* = .003), education (high school education and above) _HK_ = 45.2%, high school education and above _L_ = 32.1%; *χ*^*2*^ (1) = 14.37, *p* = .001) and BMI (BMI _HK_ = 22.02, BMI _L_ = 25.19; *t* (609) = 13.97, *p* < .001). Concerning marital status, no difference was found (*p* = .15). The majority of participants were married in both cities (see Table 1).

**Table 1:** Characteristics of overall sample and by city

	Overall	Leipzig	Hong Kong	*χ*^*2*^/*t*	*p*
Age, *M* (*SD*)	71.03 (6.87)	72.06 (6.78)	69.96 (6.81)	3.82^***^	< .001
BMI, *M* (*SD*)	23.59 (3.25)	25.19 (2.91)	22.02 (2.77)	13.97^***^	< .001
Gender, *n* (%)				8.60^**^	.003
Male	292 (47.3%)	129 (41.5%)	163 (53.3%)		
Female	325 (52.7%)	182 (58.5%)	143 (46.7%)		
Marital status, *n* (%)				2.10	.15
Single	152 (24.8%)	84 (27.3%)	68 (22.2%)		
Married	462 (75.2%)	224 (72.7%)	238 (77.8%)		
Education level, *n* (%)				14.37^***^	.001
Primary school	375 (61.7%)	205 (67.9%)	170 (54.8%)		
High school	144 (23.4%)	52 (17.2%)	92 (29.7%)		
University	93 (14.9%)	45 (14.9%)	48 (15.5%)		

*BMI* Body Mass Index;

^**^ *p* < 0.01, 2 tailed; ^***^p < 0.001, 2 tailed

### Measures of outcome and potential determinants

Self-reported questionnaires were used to investigate older adults’ personal demographics, park-based PA, psychosocial variables and perceived park environmental variables of PA. All questionnaires have been well established in previous studies and back-translated to Cantonese and German by 2 independent bilingual translators. The questionnaire items and reliability are presented as follows:

*Personal demographics*: age, gender, education (primary school, high school or university/college), marital status (single or married), height and weight.

*PBPA*: The older adults were asked to report their PA type, amount of their PA (frequency and duration per week) and intensity levels of their PA in parks during a typical week (low, moderate and vigorous). Intensity levels of PA were transferred to relevant MET values. Specifically, low, moderate and vigorous intensity were corresponded to 4 kcal/min, 6.5 kcal/min and 9 kcal/min respectively [31]. For this study energy expenditure is used as measure for PA. Energy expenditure of PBPA (kcal/week) was calculated by multiplying metabolic equivalents (MET) values (kcal/min) and time (min/week). Thus, based on the amount and the intensity of PA, energy expenditure of PBPA (kcal/week) was computed [31, 32].

*Psychosocial variables of PA in parks*: There are five psychosocial variables below. Self-efficacy was measured with the stem “I am confident that I can participate in PBPA even if ….” followed by 5 items such as “… I am tired”, “...I feel depressed” [33, 34]. Social support was measured with the stem “My family or my friends …” followed by 3 items such as “… do physical activity in park with me” [33]. Perceived benefits were measured with the stem “If I participate PA in parks, I will …” followed by 13 items such as “… feel less depressed and/or bored”, “… build up my muscle strength” [21]. Perceived barriers were measured with the stem “How often do the following reasons prevent you from getting physical active in parks?” followed by 15 items such as “Lack of time”, “Lack of good health” [21]. Enjoyment of PA was measured with the stem “Do you enjoy being physical active in parks?” followed by 3 items such as “I enjoy the feeling I get while doing PA in parks” [21]. The five-point scale ranging from 1 “don’t agree at all” to 5 “totally agree” were used for all variables above except for perceived barriers using reverse scale.

*Environmental variables of PA in parks*: There are four environmental variables below. Park safety was measured with the stem “How do you think about the safety for PA in this park?” followed by 4 items such as “In general, I feel safe in this park”, “There are no dangerous persons or behaviors (e.g. alcohol or drug use) in this park” [35]. Attractiveness of parks was measured with the stem “How do you think about the attractiveness of this park?” followed by 4 items such as “There is litter in the sidewalks in this park”, “There are beautiful trees, flowers, shrubs and wellkept grass area along the sidewalk in this Park” [36]. PA areas and features were measured with the stem “How do you think about the facilities and amenities for physical activity in this park?” followed by 4 items such as “There are sufficient facilities for physical activity in this park (e.g. field for playing balls, fitness station, open grass or path)”, “There are sufficient amenities to support physical activity in this park (e.g. benches, bathrooms or lighting)” [37]. The four-point scale ranging from 1 “don’t agree” to 4 “totally agree” were used for the three variables above. Park accessibility was measured with one item. Participants were asked to assess the time distance they took to get from home to the park with four-point scale including 1 (up to 10 min), 2 (11–20 min), and 3 (more than 20 min) [36].

Overall, the reliability of the measurements for the psychosocial and environmental variables were satisfactory (Range of Cronbach Alpha_HK_: .70–.95; Cronbach Alpha_L_: .46–.89).

### Statistical analyses

Data were analysed using SPSS 22.0. Descriptive analyses including percentages were used to present demographic differences between Hong Kong and Leipzig older adults and were examined with Chi-squared test and independent t-test. In addition, the association of demographics with energy expenditure of park-based PA were examined by t-tests, F-tests or by correlations (Pearson, Spearman). Furthermore, multiple linear regressions were used to analyse the associations between psychosocial factors and perceived environmental factors with PBPA and the moderating effect of city (urban area). To further elaborate the magnitude of the association between PBPA and factors, effect size (*f* ^2^) was calculated with the conversion equation of ${f}^2=\frac{R^2}{1-{R}^2}$. *f* ^2^ of .02 is a small effect, .15 a medium effect, and .35 a large effect [38].

## Results

### Descriptive information of PBPA of elderly, psychosocial correlates factors and perceived environmental factors of PBPA

The mean value of total energy expenditure of PBPA in the mixed-culture sample of elderly was 796.84 kcal/ week (*SD* = 689.27). Socio-demographic differences in energy expenditure are presented in Table 2. It was revealed that there were significant differences of energy expenditure in city (*t* = − 2.16, *p* < .01), gender (*t* = 3.44, *p* < .01), marital status (*t* = − 2.52, *p* < .05) and education level (*F* = 4.38, *p* < .05). In addition, Pearson correlation analyses indicated that energy expenditure was negatively correlated with BMI (*r* = − 0.09, *p* < .05) but was not correlated with age (*r* = 0.04, *p* = 0.33). The descriptive information of psychosocial factors and perceived environmental factors (Mean value and SD) are also presented in Table 2.

**Table 2:** Descriptive statistics for the associations of city and socio-demographics with energy expenditure, as well as psychosocial and perceived environmental factors in the total sample (*n* = 588–601)

	Energy expenditure of PBPA (kcal/ week)	*t* / *F*	*p*	*r*	*p*
	*M* (*SD*)				
Total	796.84 (689.27)				
City					
Leipzig	737.22 (571.22)	−2.16^*^	0.03		
Hong Kong	859.11 (790.17)				
Gender					
Male	902.84 (746.10)	3.44^**^	0.001		
Female	708.22 (613.96)				
Marital status					
Single	679.85 (672.83)	−2.52^*^	0.01		
Married	842.50 (692.86)				
Education level					
Primary	722.12^a^ (626.44)	4.38^*^	0.01		
High school	896.00^b^ (772.06)				
University	891.24^b^ (756.82)				
Age				0.04	0.33
BMI				−0.09^*^	0.04
Psychosocial factors	*M (SD)*				
Self-efficacy	*3.24 (0.91)*				
Enjoyment	*4.31 (0.81)*				
Perceived benefits	*3.78 (0.67)*				
Perceived barriers	*1.85 (0.75)*				
Social support	*2.99 (1.15)*				
Perceived park environmental factors	*M (SD)*				
Park safety	*3.41 (0.57)*				
Attractiveness	*3.25 (0.51)*				
Park features	*3.23 (0.56)*				
Park time distance	*2.14 (1.04)*				

^*^ *p* < 0.05, 2 tailed; ^**^ *p* < 0.01, 2 tailed

^a,b^: significant difference between subgroups (Duncan-test)

### Association of psychosocial factors, perceived environmental factors with PBPA of elderly

When controlling for city, gender, marital status, education level and BMI, the association of psychosocial factors and perceived park environmental factors with energy expenditure of park-based PA in the univariate regression analysis were presented in Table 3. All psychosocial factors were significantly related to the energy expenditure of PBPA, with the exception of social support. Self-efficacy, enjoyment and perceived benefits were positively associated with energy expenditure of PBPA. Perceived barriers were negatively associated with the energy expenditure. In terms of the perceived park environmental predictors, perception of park safety, park attractiveness and park features had a positive association with energy expenditure of PBPA, but perceived park time distance had a negative association with energy expenditure.

**Table 3:** Results of univariate regressions between psychosocial factors, park environmental factors and PBPA energy expenditure (*n* = 526–569)

Correlates	*B* (SE)	*β*	95% CI	Adjusted *R*^*2*^
Psychosocial factors				
Self-efficacy	200.84 (30.77)	.27^***^	[140.40, 261.28]	.11^***^
Enjoyment	271.80 (38.76)	.32^***^	[187.60, 365.06]	.11^***^
Perceived benefits	222.42 (41.82)	.22^***^	[140.28, 304.57]	.08^***^
Perceived barriers	− 458.56 (45.14)	−.48^***^	[− 547.22, − 369.90]	.20^***^
Social support	31.16 (25.26)	.05	[−18.46, 80.78]	.04^***^
Perceived park environmental factors				
Park safety	103.89 (50.60)	.09^*^	[4.50, 203.27]	.05^***^
Attractiveness	167.28 (59.99)	.12^**^	[49.45, 285.12]	.05^***^
Park features	103.20 (50.46)	.09^*^	[4.09, 202.31]	.05^***^
Park time distance	−73.63 (27.64)	−.11^**^	[− 127.90, −19.36]	.05^***^

Control variables included city, gender, marital status, education level and BMI.

^*^ *p* < 0.05, 2 tailed; ^**^ *p* < 0.01, 2 tailed; ^***^p < 0.001, 2 tailed

### City moderating the associations of factors with park-based PA of elderly

Except city, significant demographic variables (gender, marital status, education level), BMI and significant psychosocial factors revealed in univariate analyses were first entered as independent variables in Model 1 (see Table 4). The linear combination of gender, education level, BMI, self-efficacy, perceived benefits and perceived barriers scores significantly predicted energy expenditure, *R*^*2*^ = 0.18,*F* (6, 518) = 13.76, *p* < .001 (see Table 4). The significant perceived environmental factors revealed in univariate analyses were entered in Model 2 (see Table 4). Only perceived park time distance significantly contributed to the model, *R*^*2*^ change = 0.01, *F* (12, 518) = 10.16, *p* < .001 (see Table 4). City was entered in Model 3 and significantly contributed to this model, *R*^*2*^ change = 0.06, *F* (13, 518) = 13.02, *p* < .001 (see Table 4). Finally, the interaction between city, psychosocial factors and perceived park environmental factors were entered in Model 4. Terms for the interactions between city and park features as well as between city and park time distance significantly contributed to the model, *R*^*2*^ change = 0.02, *F* (21, 518) = 8.94, *p* < .001 (see Table 4). The full model (Model 4) eventually accounted for 27% of variance in energy expenditure. In addition, the effect size (f ^2^) of association for each model indicated that Model 1 f ^2^ = 0.22, Model 2 f ^2^ = 0.23, Model 3 f ^2^ = 0.33 and Model 4 f ^2^ = 0.37, suggesting the large effect of association (f ^2^ > 0.35) was in full model (Model 4).

**Table 4:** Multiple regression results for prediction of PBPA energy expenditure (*n* = 526)

Variables	Model 1		Model 2		Model 3		Model 4	
	*B* (95% CI)	*β*	*B* (95% CI)	*β*	*B* (95% CI)	*β*	*B* (95% CI)	*β*
Gender	87.50 [33.60, 141.39]	.13^**^	82.28 [28.47, 136.09]	.12^**^	53.66 [0.93, 106.39]	.08^*^	50.89 [−2.00, 103.79]	.08
Marital status	−4.35 [−67,61, 58.92]	−.01	− 2.53 [−65.66, 60.59]	−.003	28.05 [−33.65, 89.76]	.04	25.52 [− 36.38, 87.42]	.03
Education level^a^	73.51 [16.79, 130.23]	.11^*^	86.41 [29.13, 143.68]	.12^**^	84.94 [29.65, 140.24]	.12^**^	89.05 [33.76, 144.34]	.13^**^
BMI	−36.77 [−54.01, −19.53]	−.18^***^	− 33.11 [−50.55, −15.68]	−.16^***^	−7.86 [−26.50, 10.78]	−.04	−8.43 [− 26.78, 10.13]	−.04
Self-efficacy	114.43 [47.87, 180.98]	.16^**^	106.41 [39.58, 173.24]	.15^**^	109.57 [45.05, 174.10]	.15^**^	97.77 [32.29, 163.24]	.13^**^
Enjoyment	−2.84 [−95.71, 90.03]	−.003	0.23 [−93.79, 94.25]	.000	73.91 [−19.81, 167.63]	.09	50.64 [−44.81, 146.09]	.06
Perceived benefits	114.46 [23.12, 205.80]	.11^*^	111.98 [19.95, 204.00]	.11^*^	59.72 [−30.64, 150.10]	.06	98.79 [1.76, 195.82]	.10
Perceived barriers	− 219.60 [−318.32, −120.87]	−.23^***^	− 238.86 [− 341.23, −136.50]	−.25^***^	− 344.94 [− 449.32, − 240.56]	−.36^***^	− 323.82 [− 447.65, − 200.00]	−.34^***^
Safety			−7.56 [−114.94, 99.83]	−.01	5.82 [−97.94, 109.58]	.01	−14.77 [− 119.64, 90.11]	.01
Attractive-ness			−109.43 [− 244.06, 25.20]	−.08	−61.75 [− 192.59, 69.10]	−.05	−31.06 [−163.90, 101.77]	−.02
Park features			108.28 [−3.83, 220.39]	.09	48.16 .[−61.73, 158.06]	.04	43.42 [−68.97, 155.81]	.04
Park time distance			−62.96 [− 115.62, −10.29]	−.10^*^	−34.21 [−85.87, 17.44]	−.05	−24.17 [− 7676, 28.42]	−.04
City					233.77 [159.69, 307.84]	.35^***^	224.15 [143.34, 304.96]	.33^***^
City * Self-efficacy							42.50 [−22.19, 107.18]	.06
City * Enjoyment							0.22 [−95.13, 95.60]	.000
City * Perceived benefits							66.74 [−30.17, 163.66]	.07
City * Perceived barriers							−28.47 [−150.60, 93.66]	−.03
City* safety							7.43 [−96.95, 111.82]	.01
City * Attractiveness							−35.68 [− 168.73, 97.37]	−.03
City * Park feature							−119.83 [− 230.71, −8.95]	−.10^*^
City * Park time distance							63.62 [11.12, 116.12]	.10^*^

Model 1 *R*^*2*^ = .18; Model 2 *R*^*2*^ = .19; Model 3 *R*^*2*^ = .25; Model 4 *R*^*2*^ = .27

*Effect size (f* ^*2*^*) of association: Model1 f* ^*2*^ *= 0.22; Model 2 f* ^*2*^ *= 0.23; Model 3 f* ^*2*^ *= 0.33; Model 4 f* ^*2*^ *= 0.37*

^a^Education level was divided into two categories: low level (primary school) and middle to high level (high school and university)

* *p* < 0.05, 2 tailed, ** *p* < 0.01, 2 tailed, ***p < 0.001, 2 tailed

**Fig. 1:**
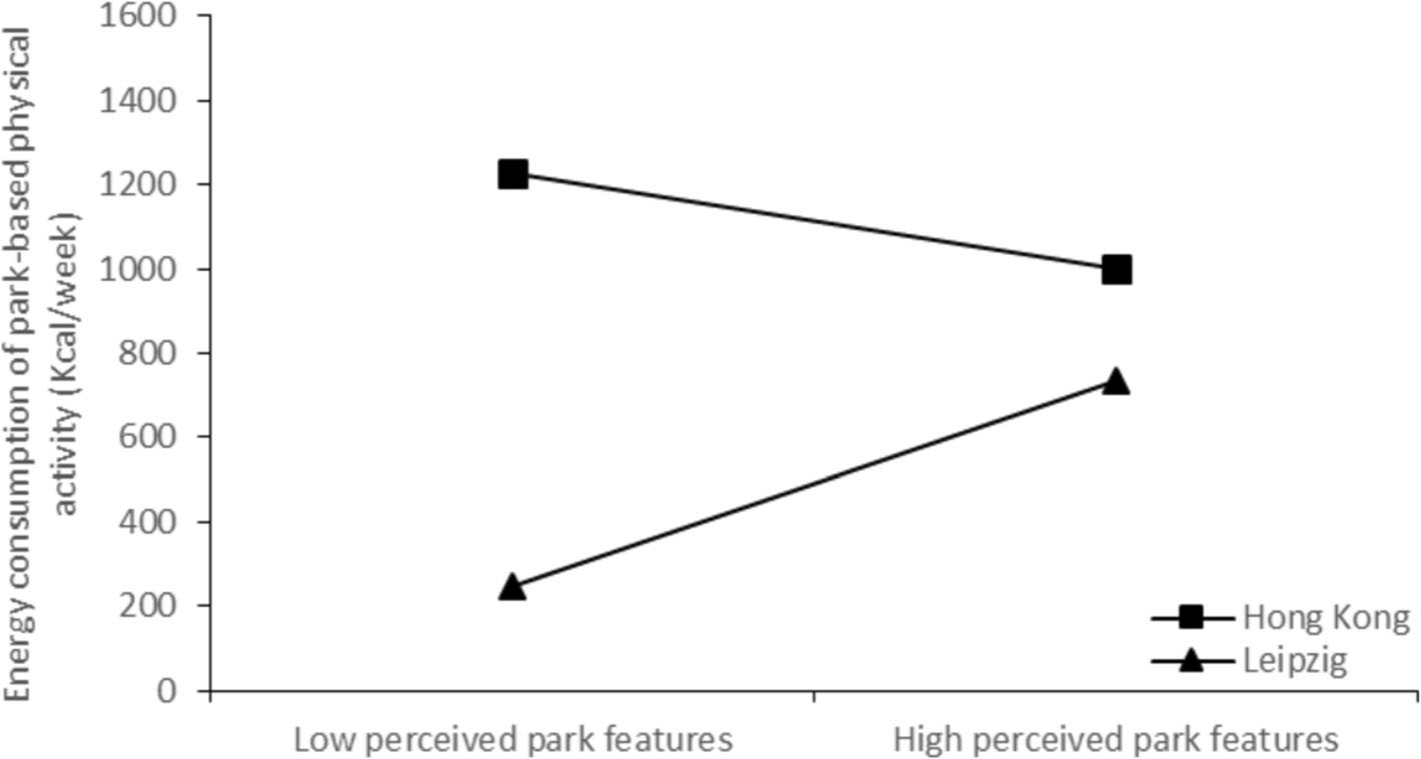
Regression lines for perceived park features and PBPA energy expenditure, moderated by city

**Fig. 2:**
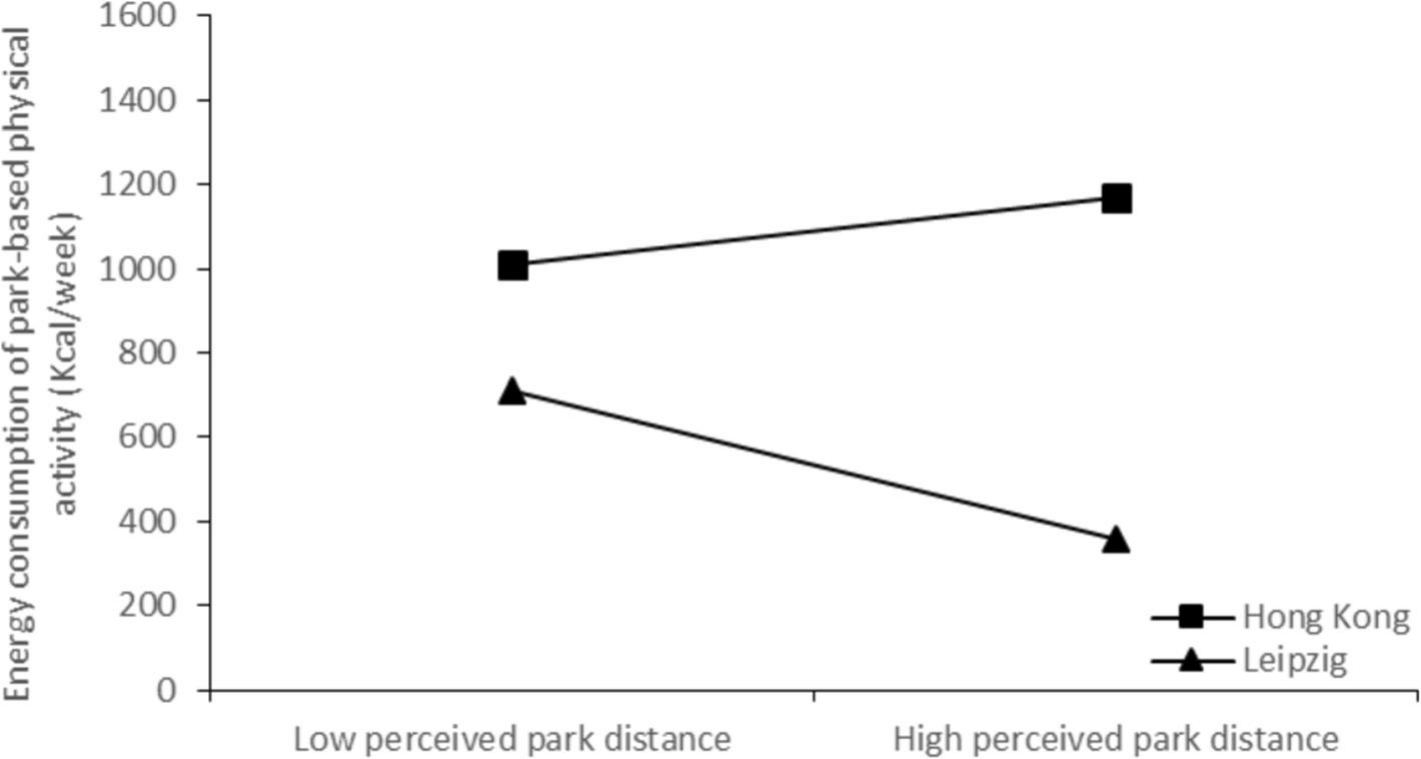
Regression lines for perceived park time distance and PBPA energy expenditure, moderated by city

To further explore these interaction terms, simple slopes analyses were conducted to examine the moderating effect of city on the associations of perceived park features and perceived park time distance with energy expenditure. As shown in Fig. 1, there was no significant relationship between perceived park features and energy expenditure in Hong Kong [*β* = −.05, *t* (253) = − 0.77, *p* = .44], whereas a significant positive association was found in Leipzig [*β* = .15, *t* (271) = 2.14, *p* = .03]. As indicated in Fig. 2, there was no significant relationship between perceived park time distance and energy expenditure in Hong Kong [*β* = .05, *t* (253) = 0.83, *p* = .41], whereas a significant negative association was found in Leipzig [*β* = −.17, *t* (271) = − 2.93, *p* = .004].

## Discussion

This study was conducted to examine the association of psychosocial factors and perceived park environmental factors with PBPA of elderly and to evaluate the moderating effect of urban area (city) on the associations of factors with PBPA of elderly.

Findings of the current study revealed that there are differences between Hong Kong and Leipzig, referring to the self-reported PBPA. The energy expenditure of PBPA of all the elderly in the parks was higher in Hong Kong than in Leipzig. This might be explained by the particular urban conditions in Hong Kong compared to Leipzig. In Hong Kong the climate is warmer and the population density is higher. As described in Hong Kong there is a greater number of high-rise buildings and various opportunities to come into contact with nature. These reasons were evident also in other research. Klenk et al. [39] analysed the PA of elderly in the “Walking on Sunshine” study. The walking times outside depend on weather conditions. In addition, cultural factors might come into play: Germans traditionally join sport clubs or gymnastic clubs [40] and are more likely to engage in PA, whereas, in Hong Kong making use of parks to engage in PA has been a long-standing Chinese cultural tradition [9]. In addition, the BMIs of the samples in both cities (Leipzig: 25.19; Hong Kong: 22.02) is lower for the group of elderly aged 60 and above in Germany (28.6, [41]) and Hong Kong (24.12, [42]), showing that PBPA is an element of a healthy lifestyle in both cities. The negative association between energy expenditure of PBPA and BMI is also consistent with previous studies [9, 10, 43].

The current study demonstrates consistent further results revealing that differences in energy expenditure of elderly, in both cities, is related also to other demographic variables [12]. Men have a higher energy expenditure during PBPA than women, married older adults are more active than singles and the energy expenditure during PBPA of elderly with a high education level is higher than for older adults with a lower education level.

Regarding psychosocial factors it can be stated that four out of five psychosocial variables are significantly associated with energy expenditure in PBPA of older adults, including self-efficacy, perceived barriers, benefits of PA, enjoyment of PA – but not social support. Except for the latter, these findings for PBPA are in line with previous literature across other different fields and conditions of PA [12, 15, 44]. Regarding the association of social support and PA there are different results in the literature. For the adoption of PA, social support is a significant predictor, for the maintenance of PA of older adults, enjoyment of PA and social networks seem to be more important [12, 45].

For promoting PBPA in the elderly such knowledge about psychosocial correlates might be helpful. For example, in order to enhance self-efficacy, the creation of success experiences and positive feedback or substitute reinforcement [46] are recommended. For perceived barriers, currently various techniques for influencing planning and management of perceived barriers are discussed in the literature [47, 48], but having knowledge about the concrete crucial barriers is the precondition to overcome them. A study of Devereux-Fitzgerald and colleagues [49] systematically reviewed the qualitative studies, which investigated the specific needs of older adults who adopted and adhered to sports and exercise programmes. These needs in turn can be regarded as barriers in the case of non-compliance. Thus, for older people intensive and competent care during PA and exercise is more important than for younger. They also want the instructor to be knowledgeable about the elderly and their common health conditions. In addition, the purpose and benefits of physical activity must be clear and the information has to be transparent [49].

In view of the environmental conditions in which physically active older adults practice their PBPA, the selected four perceived environmental correlates in this study are significantly associated with the self-reported PBPA of the elderly. So, the results for the park safety, attractiveness, park features and park time distance are also consistent with previous studies [16] and can also apply to the elderly. According to the realist synthesis of Yen et al. [25] the mobility of older adults is decisively influenced by the estimated safety of their residential area with its various movement areas such as parks, pedestrian and cycle paths, their furnishing or design as well as the presence of further infrastructure. The study of Bethancourt, Rosenberg, Beatty and Arterburn [50] provides hints, that it is a great barrier for the adoption of PA of elderly to have an unsafe, uninviting, unattractive environment or uneven paths.

Based on the current study findings regarding the association of psychosocial correlates and perceived park environmental correlates with PBPA, the results will be inspiring and informative for future PA intervention design for older adults in parks. Health promoters can particularly help the elderly to gain higher selfconfidence during PA, find more enjoyment in PA and improve perceived health benefits of PA by offering park-based programmes. In terms of environmental factors, park designers should consider a high park security, attractive features like sufficient PA facilities with good quality, and amenities that support elderly during PA. In addition, PA in the elderly is encouraged if the park is within close proximity or accessible distance of their residence or home. These aspects could also be shown in the associated observa-tional study [27].

The elderly’s energy expenditure was also positively related to city, which in our study stands for different geographical locations with different urban areas [28]. City was one of the strongest predictors with an own contribution to explain park-based energy expenditure of the elderly. As with many results of studies from other countries [51, 52], the conditions for PA in Asia (or China) may not be 1:1 transferable to Europe. For example, the places and structures in which physical activities are executed in Hong Kong are fundamentally different from German urban structures.

There are significant interactions of city with perceived park feature and perceived park time distance concerning park-based energy expenditure PA of elderly. In Hong Kong perceived park features seemed to have no significant importance for PA. In contrast, elderly in Leipzig who perceived higher park features are more likely to engage in PBPA compared with those who perceive lower park features. That means, if park features in Leipzig have a higher quality, the elderly are likely to do more PA. The possible reason for the different association among the two cities might be, that in Hong Kong a park is the “main” place to execute PA for elderly irrespective of the quality of the park features. In Leipzig the elderly also have the opportunity to do PA elsewhere, such as public sport clubs or commercial health and fitness centers. This explanation is also in line with the results for the perceived park time distance. In Hong Kong, there were no significant differences in perceived park time distance for PA energy expenditure. That means again older people in Hong Kong are satisfied because they have parks with multiple functions as the “main” place to be active, irrespective of perceived park time distance.

In Leipzig elderly who perceived lower park distance are more likely to engaged in PBPA compared to those who perceive higher park distance. This result is consistent with other findings on the importance of perceived environmental conditions in Europe. Van Dyck et al. [28] also reported differences for the park users because of the neighborhood walkability. Additionally, in Hong Kong, parks probably play a more significant role in providing an attractive environment for elderly to participate in PA. In Leipzig, parks are possible perceived more as a place to relax or walk instead of a place to do exercise and be vigorously active [27]. To attract the park areas for PA of elderly and to promote the PA engagement of elderly these results of different associations of perceived park features and perceived park time distance with PBPA of elderly should be considered by park planners and policy makers.

The present study has several strengths. The data were measured with valid and reliable questionnaire tools, translated into three languages (English, German, Cantonese). An identical study protocol was used in Hong Kong and Leipzig. Therefore, it is possible to compare the findings of two cities with different urban areas in different countries. These comparisons revealed significant differences about park users and park characteristics, especially in addition with a direct observation [27]. However, study limitations also need to be acknowledged. Cross-sectional analyses make it impossible to infer causal relationships between PBPA and the relevant attributes, including the psychosocial and perceived park environmental factors. The questionnaires used in this study to measure PBPA in older people were validated. Nevertheless, objective and direct measurement, for example with accelerometers, is still required to accurately measure the energy expenditure of PBPA of elderly in the parks [53]. Moreover, in addition to the difference of urban area (high building and population density vs. low building and population density) in two cities, culture difference exisits as well. There is a need to make specific environmental factors of cities and parks and there operationalization more concrete. Regarding the older adults who participated in the study, only limited information of park use was collected, making it impossible to know if they lived in the neighborhood around the parks or not. Of further concern, this study did not consider the impacts of selection bias on older adults’ park-based PA because of the cross-sectional nature of the current study. Selection bias may occur when active individuals choose to live in the places that are equipped with active ressources [38]. The bias in selection is likely to bring an over estimation of park environment because active older adults with the choosen place to live may consider parks as a resource for maintaining an active lifestyle.

## Conclusion

In conclusion, the findings of the current study are profound, as there is indication that selected psychosocial and environmental factors are associated with PBPA of elderly, even in a mixed sample from two cities. Furthermore, city as urban area has an independent contribution to park-based PA of the elderly. City moderates the associations of these factors with PBPA of elderly in the way that, for elderly, parks in Hong Kong are more relevant for PA, irrespective of park features or perceived park time distance. In Leipzig, a city with a different urban structure, perceived park features and perceived park time distance are relevant for the PA of elderly in parks. Therefore, the interaction of perceived environmental factors and PBPA in different urban areas should be considered in more detail in the future. Park designers and policy makers on the municipal level could use the findings to choose adequate strategies to promote PA of elderly. In order to make parks more attractive, targeted interventions on adequate equipment and accessibility for older people could be particularly effective to promote park-bases PA of the elderly.

## Data Availability

Requests for data and material should be directed to the study director, Prof. Dr. Petra Wagner (petra.wagner@uni-leipzig.de), and/or Dr. Yan Ping Duan (duanyp@hkbu.edu.hk).

## Abbreviations

BMI: Body mass index
e.g.: For example
ha: Hectare
HK: Hong Kong
kcal: Kilocalorie
km: Kilometre
L: Leipzig
PA: Physical activity
PBPA: Park-based physical activity
